# Prognostic Values of METTL3 and Its Roles in Tumor Immune Microenvironment in Pan-Cancer

**DOI:** 10.3390/jcm12010155

**Published:** 2022-12-25

**Authors:** Yang Guo, Yu Heng, Hui Chen, Qiang Huang, Chunping Wu, Lei Tao, Liang Zhou

**Affiliations:** Department of Otorhinolaryngology Head and Neck Surgery, Shanghai Key Clinical Disciplines of Otorhinolaryngology, Eye and ENT Hospital of Fudan University, 83 Fenyang Road, Xuhui District, Shanghai 200031, China

**Keywords:** METTL3, pan-cancer, biomarker, prognosis, tumor microenvironment

## Abstract

**Background:** N6-methyladenosine (m^6^A) is among the most prevalent RNA modifications regulating RNA metabolism. The roles of methyltransferase-like 3 (METTL3), a core catalytic subunit, in various cancers remain unclear. **Methods:** The expression levels of METTL3 in pan-cancer were profiled and their prognostic values were examined. We assessed the relationships between METTL3 expression levels and tumor immune infiltration levels, immune checkpoint gene expression, immune neoantigens, tumor mutation burden, microsatellite instability, and DNA mismatch repair gene expression. Furthermore, a protein–protein interaction network was drawn, and gene set enrichment analysis was conducted to explore the functions of METTL3. **Results:** METTL3 expression levels were elevated in most cancers, with high expression associated with poorer overall and disease-free survival. METTL3 levels were significantly related to immune cell infiltration, tumor mutation burden, microsatellite instability, mismatch repair genes, and immune checkpoint gene levels. METTL3 was enriched in pathways related to RNA modification and metabolism and correlated with epithelial–mesenchymal transition. **Conclusions:** METTL3 serves as an oncogene in most cancer types and shows potential as a prognostic biomarker. Additionally, our comprehensive pan-cancer analysis suggested that METTL3 is involved in regulating the tumor immune microenvironments and epithelial–mesenchymal transition via modulating RNA modification and metabolism, making it a potential therapeutic target.

## 1. Introduction

Methylated adenosine at the *N*^6^ position, named as N^6^-methyladenosine (m^6^A), is among the most abundant internal modifications within RNA [1]. m^6^As are deposited by methyltransferases (“writers”) and removed by demethylases (“erasers”), the balance of which is dynamically regulated to ensure that normal bioprocess occur in various human organs and tissues [1]. Although m^6^A modifications do not directly affect base pairing and coding abilities, they can affect the expression levels of genes at multiple levels by modulating the balance between oncogenes and cancer suppressor genes to participate in tumor development and progression [2,3,4,5].

As a pathologically active niche that shapes tumor evolution, the tumor microenvironment (TME) plays a crucial role in therapy resistance and immune escape [6]. Dysregulated m^6^A modifications contribute to the heterogeneity and complexity of TMEs [7]. Studies have suggested that m^6^A modification can modulate epigenetic modifications of immune-related genes with essential roles in the TME [8,9,10,11]. In addition, m^6^A modification can regulate the proliferation, activation, and function of immune cells, as well as the development and functions of other stromal cells in the TME [12,13,14,15]. 

METTL3, the sole catalytic core in methyltransferase complex, uses *S*-adenosylmethionine as the methyl donor [16] and is an essential regulator of the activities of various immune cells [13]. Tumor-infiltrating immune cells in the TME are potential biomarkers of the effectiveness of immunotherapy and disease prognosis of different cancers [17]. Therefore, the role of METTL3 in the tumor immune microenvironment warrants further investigation.

This study was conducted to analyze the expression profiles of METTL3 and its prognostic value in pan-cancer. The results suggest that METTL3 mainly acts as an oncogene and can serve as a prognostic biomarker in pan-cancer. The significant associations between METTL3 expression and immune factors in the TME suggest that METTL3 plays important roles in the tumor immune microenvironment and that therapeutic approaches can be developed to target METTL3.

## 2. Materials and Methods

### 2.1. Expression Analysis of METTL3 and Genomic Alterations of METTL3 in Pan-Cancer and Cancer Cell Lines

The expression profiles of METTL3 across all The Cancer Genome Atlas (TCGA) tumor data (Supplementary Material Table S1) were presented in box plots using TIMER [18]. The mRNA expression levels of METTL3 in various cancer cell lines were obtained from the Cancer Cell Line Encyclopedia (CCLE; https://www.broadinstitute.org/ccle, accessed on 1 May 2021) database, and the data were presented as box plots. The online cBioPortal database (http://www.cbioportal.org/, accessed on 1 May 2021) for cancer genomics was used to explore alterations in the METTL3 status in different cancers. 

### 2.2. Prognostic Value of METTL3 in Pan-Cancer

Using the Gene Expression Profiling Interactive Analysis server, survival curves in patients with different expression levels of METTL3 were plotted for various types of cancers. Additionally, the associations of METTL3 expression levels with diverse pathological stages were explored based on TCGA clinical annotation.

### 2.3. Relationships between Expression Levels of METTL3 and Immune Infiltrating Levels, Immune Checkpoint Gene Levels, Immune Neoantigens, Tumor Mutational Burden, Microsatellite Instability, and DNA Mismatch Repair Genes in Pan-Cancer

TIMER was used to explore the molecular characterization of tumor-immune interactions as described previously [18]. The relationships between the expression of METTL3 and that of various immune checkpoint genes and the number of neoantigens were analyzed as described in previous studies [19,20]. The relationships between the expression levels of METTL3 and microsatellite instability (MSI), tumor mutation burden (TMB), and mismatch repair (MMR) genes in pan-cancer were also assessed.

### 2.4. Protein–Protein Interaction Network, Gene Ontology, and Kyoto Encyclopedia of Genes and Genomes Enrichment Analysis and Gene Set Enrichment Analysis of METTL3

GeneMANIA was used to build a protein–protein interaction (PPI) network of METTL3. Gene Ontology (GO) and Kyoto Encyclopedia of Genes and Genomes (KEGG) enrichment analyses of the proteins were conducted using the R package clusterProfiler [21]. Gene set enrichment analysis (GSEA) was used to obtain an ordered list of genes related to the expression levels of METTL3. Only the top five KEGG and hallmark terms were identified. The results of enriched pathways were considered as significant based on a threshold of |normalized enrichment score| > 1, *p*-value < 0.05, and false discovery rate < 0.25.

### 2.5. Validation of Expression Profiles of METTL3 in Gene Expression Omnibus and Clinical Tissues

The expression levels of METTL3 were analyzed in the GSE87410 dataset, which consisted of nine types of cancers: cervical squamous cell carcinoma and endocervical adenocarcinoma (CESC), esophageal carcinoma (ESCA), stomach adenocarcinoma (STAD), liver hepatocellular carcinoma (LIHC), lung adenocarcinoma (LUAD), lung squamous cell carcinoma (LUSC), thyroid carcinoma (THCA), small-cell lung carcinoma (SCLC), and gastric signet-ring cell carcinoma (SRCC) [22]. 

Head and neck squamous cell carcinoma (HNSC) tissues and matched adjacent normal tissues were collected from patients undergoing surgery at the Department of Otorhinolaryngology-Head and Neck Surgery of the Eye and ENT Hospital of Fudan University. The expression levels of METTL3 were validated using quantitative reverse transcription-polymerase chain reaction as described previously in 25 pairs of HNSC tissues and normal tissues [23]. Further immunohistochemistry analysis and Western blot analysis were conducted in pairs of HNSC tissues and normal tissues as previously described [24]. The experiments were approved by the Ethics Committee of the Eye and ENT Hospital of Fudan University and were conducted according to established ethical guidelines. All subjects provided written informed consent prior to sample collection. 

### 2.6. Statistical Analysis

The Wilcoxon test was used to explore the expression profiles of METTL3 in tumor tissues and normal tissues, Kaplan–Meier analyses were conducted to compare the differences between the survival times of the high- and low-METTL3-expression groups. Spearman correlation analyses were performed to explore the relationships between METTL3 expression levels and immune cell infiltration levels, TMB, MSI, MMR genes, and immune checkpoint genes. Statistical significance was set at *p* < 0.05.

## 3. Results

### 3.1. Upregualted Expression of METTL3 in Pan-Cancer Tissues and Cell Lines and Its Different Genetic Alterations in Cancers

**Figure 1:**
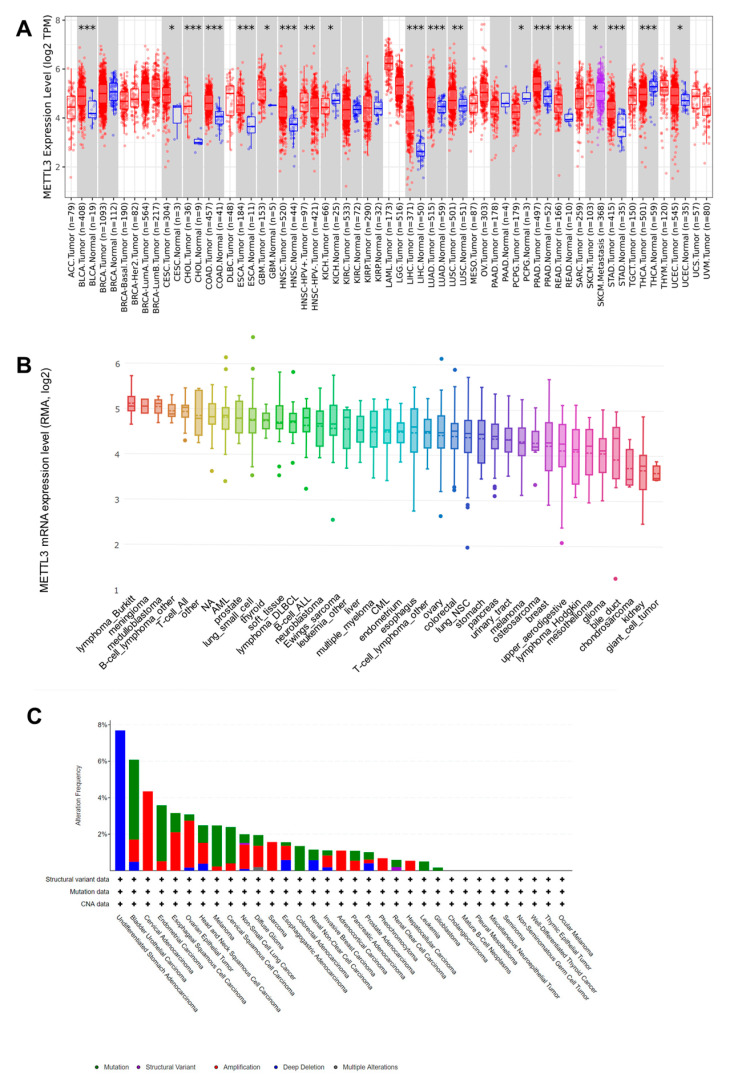
(**A**) Expression levels of METTL3 in pan-cancer determined using the TIMER database (* *p* < 0.05, ** *p* < 0.01, *** *p* < 0.001). (**B**) Expression of METTL3 in pan-cancer cell lines based on the CCLE database. (**C**) Genomic alteration type and frequency of METTL3 in various cancers (green: mutation; purple: structural variant; red: amplification; blue: deep deletion; grey: multiple alterations).

By analyzing the TIMER database, the expression profiles of METTL3 across various cancer types and their corresponding normal tissues were determined (Figure 1A). The expression levels of METTL3 mRNA were elevated in most cancers, including BLCA, CESC, CHOL, COAD, ESCA, GBM, HNSC, LIHC, LUAD, LUSC, PRAD, READ, STAD, and UCEC. Notably, HNSC with a human papillomavirus-positive status showed higher levels of METTL3 compared to that in HNSC with a human papillomavirus-negative status. Additionally, SKCM with metastasis exhibited higher levels of METTL3 compared to that of SKCM without metastasis. In contrast, the expression levels of METTL3 mRNA were downregulated in KICH, PCPG, and THCA.

We next explored the mRNA expression of METTL3 in various cancer cell lines using the CCLE database. The expression levels of METTL3 in lymphoma_Burkitt, meningioma, medulloblastoma, B-cell_lymphoma_other, and T cell_ALL were higher than those in other tumor types (Figure 1B). 

The development and progression of malignant tumors are associated with gene mutations. We found that deep deletion of METTL3 was most prominent in undifferentiated stomach adenocarcinomas, whereas its amplification was dominant in cervical adenocarcinomas, ESCA, ovarian epithelial tumor, HNSC, non-small cell lung cancer, diffuse glioma, SARC, esophagogastric adenocarcinoma, invasive breast carcinoma, ACC, pheochromocytoma, and LIHC (Figure 1C). Mutation of METTL3 was more frequently observed in BLCA, endometrial carcinoma, melanoma, cervical squamous cell carcinoma, COAD, leukemia, and GBM. 

### 3.2. METTL3 Expression Is Associated with Prognosis in Pan-Cancer

**Figure 2:**
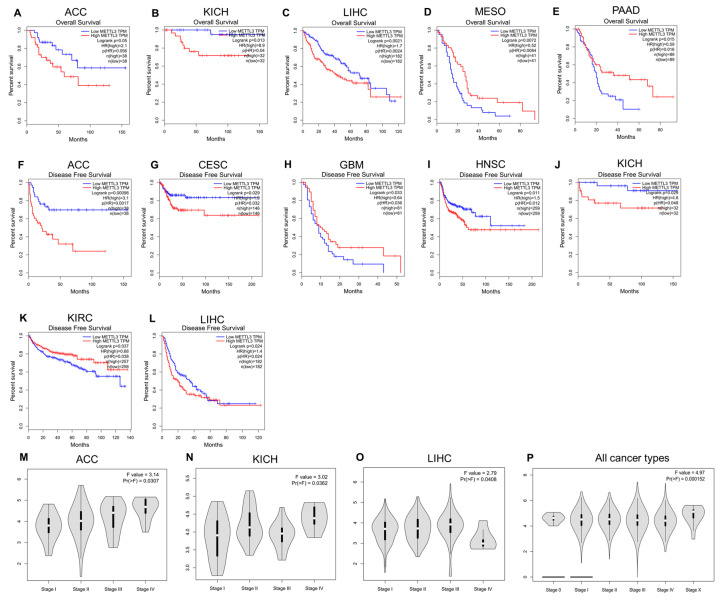
(**A**–**E**) Association between METTL3 expression levels and overall survival of patients with ACC, KICH, LIHC, MESO and PAAD. (**F**–**L**) Association between METTL3 expression levels and disease-free survival (DFS) of patients with ACC, CESC, GBM, HNSC, KICH, KIRC and LIHC. (**M**–**P**) Association between METTL3 expression levels and tumor stages of patients with ACC, KICH, LIHC and pan-cancer.

As shown in Figure 2A–E, higher expression levels of METTL3 were significantly associated with poorer overall survival in ACC, KICH, and LIHC, whereas they were significantly associated with better overall survival in MESO, and PAAD. 

Furthermore, the association between METTL3 expression levels and disease-free survival (DFS) in patients with cancer was explored (Figure 2F–L). The results indicate that higher levels of METTL3 were significantly associated with poorer DFS in ACC, CESC, HNSC, KICH, and LIHC but were significantly associated with better DFS in GBM and KIRC. Thus, dysregulated expression of METTL3 is an important factor affecting the survival of patients with cancer. However, the specific roles of METTL3 in various cancers are diverse and should be further explored in detail. 

To explore the mechanism of METTL3 in the above cancers, the relationship between the expression levels of METTL3 and tumor stages was investigated (Figure 2M–P). METTL3 expression levels were higher in patients in later stages of ACC and KICH; however, the expression levels of METTL3 in patients with LIHC with stage IV cancers were significantly lower than those in patients with LIHC at earlier stages. In addition, when the data for all patients with cancer were combined, the results suggested that METTL3 expression levels were significantly higher in later stages than in earlier stages of disease.

### 3.3. METTL3 Expression Is Correlated with Immune Infiltrating Levels in Pan-Cancer

The correlations between the expression levels of METTL3 and immune infiltrating levels in cancer were explored using the TIMER database. METTL3 levels were significantly correlated with more types of immune infiltrating cells in LIHC, LUAD, PAAD, PRAD, SKCM, and THCA. These results indicate that METTL3 plays an important role in the tumor immune microenvironment (Supplementary Material Figure S1). 

### 3.4. METTL3 Expression Is Correlated with Immune Checkpoint Gene Levels and Immune Neoantigen Loads in Pan-Cancer

**Figure 3:**
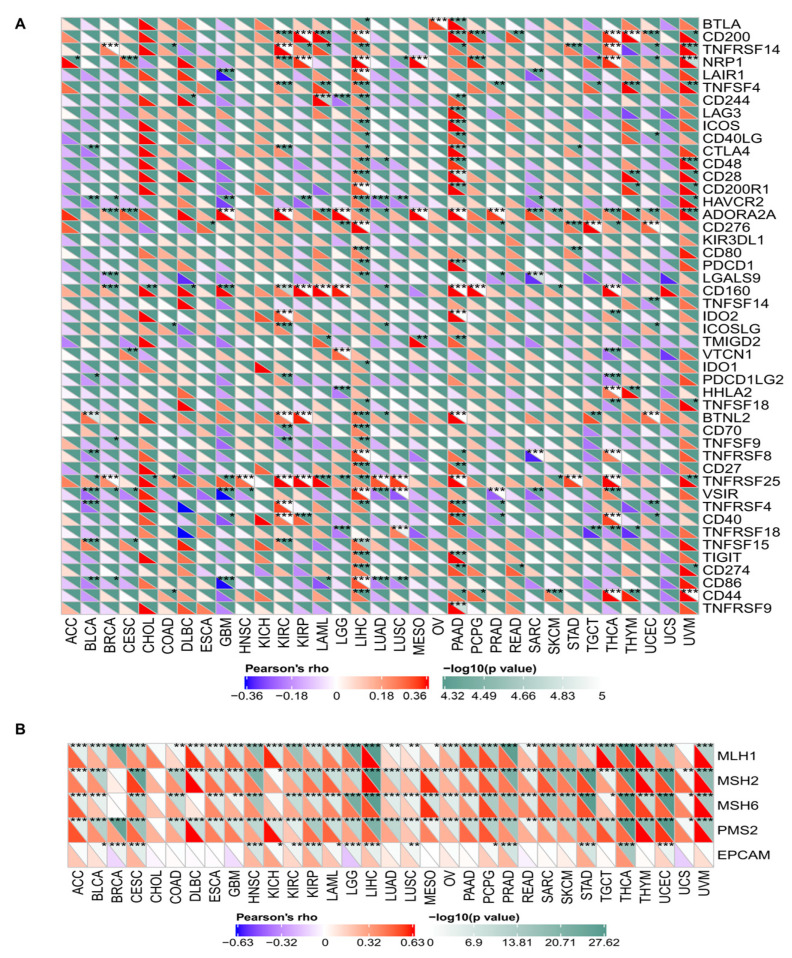
(**A**) Correlation analysis between expression levels of METTL3 and expression levels of immune checkpoint genes in pan-cancer (* *p* < 0.05, ** *p* < 0.01, *** *p* < 0.001). (**B**) Correlation analysis between METTL3 expression and mismatch repair (MMR) gene expression (* *p* < 0.05, ** *p* < 0.01, *** *p* < 0.001).

The relationships between the expression levels of METTL3 and those of immune checkpoint genes are shown in Figure 3A. Most immune checkpoint genes evaluated were positively correlated with the expression levels of METTL3. In LIHC and PAAD, the number of immune checkpoint genes significantly positively correlated with METTL3 expression, which was the highest among different cancers. The immune checkpoint genes *TNFRSF25* and *ADORA2A* were positively correlated with METTL3 expression in most cancer types. In contrast, *VSIR*, *HAVCR2*, and *CD86* were negatively correlated with METTL3 expression in several cancer types.

The relationships between METTL3 expression and tumor neoantigens in pan-cancer are presented in Supplementary Material Figure S2. In BRCA, CESC, and THCA, the expression levels of METTL3 were significantly negatively correlated with the number of tumor neoantigens. However, the expression levels of METTL3 were significantly positively correlated with the number of tumor neoantigens in STAD, HNSC, and PRAD.

### 3.5. METTL3 Expression Is Correlated with TMB, MSI, and DNA MMR Genes in Pan-Cancer

METTL3 expression levels were positively correlated with the TMB in COAD, GBM, SKCM, and STAD but negatively correlated with the TMB in BRCA, KIRC, THCA, and UVM (Supplementary Material Figure S3A). As shown in Supplementary Material Figure S3B, METTL3 expression levels were positively correlated with MSI in CESC, HNSC, KICH, LGG, LUAD, LUSC, OV, PRAD, SARC, STAD, THCA, and UCEC, but negatively correlated with the MSI in DLBC. 

The relationships between the expression level of METTL3 and those of five DNA MMR genes were analyzed (Figure 3B). Correlation analyses showed that the expression levels of METTL3 were positively associated with those of DNA MMR genes in almost all types of cancers. 

### 3.6. METTL3 Is Enriched in Pathways Related to RNA Modification and Metabolism as Well as EMT 

To examine the functions and underlying molecular mechanisms of METTL3 in various tumors, a PPI network of METTL3 was constructed using GeneMANIA (Supplementary Material Figure S4). The most noticeable protein in the network was METTL14, which was connected to METTL3 via a physical interaction and was predicted to have a functional relationship. Additionally, METTL3 physically interacts with WTAP and RBM15B. 

GO and KEGG enrichment analyses were conducted to determine the roles of METTL3 and the genes involved in the PPI network. As shown in Supplementary Material Figure S5, METTL3 and its PPI network members were mainly enriched in pathways related to RNA polymerase, mRNA surveillance pathway and RNA transport. The results of GO analysis showed that genes were enriched in RNA splicing, mRNA processing, RNA capping, and fibroblast growth factor receptor signaling pathway in the Biological Process category; in DNA-directed RNA polymerase complex, RNA polymerase complex, nuclear speck, methyltransferase complex in the Cellular Component category; and in catalytic activity acting on RNA, mRNA binding, methyltransferase activity, transferase activity transferring one-carbon groups, chromatin binding, and RNA methyltransferase activity in the Molecular Function category.

GSEA was conducted to identify dysregulated pathways between groups with high and low expression of METTL3 (Supplementary Material Figure S6). The hallmark enrichment results suggest that EMT is significantly associated with METTL3 expression. The KEGG enrichment results suggest that no pathways are significantly associated with METTL3 expression.

### 3.7. Upregulation of METTL3 Validated Using the GEO Database and Clinical Tissues

**Figure 4:**
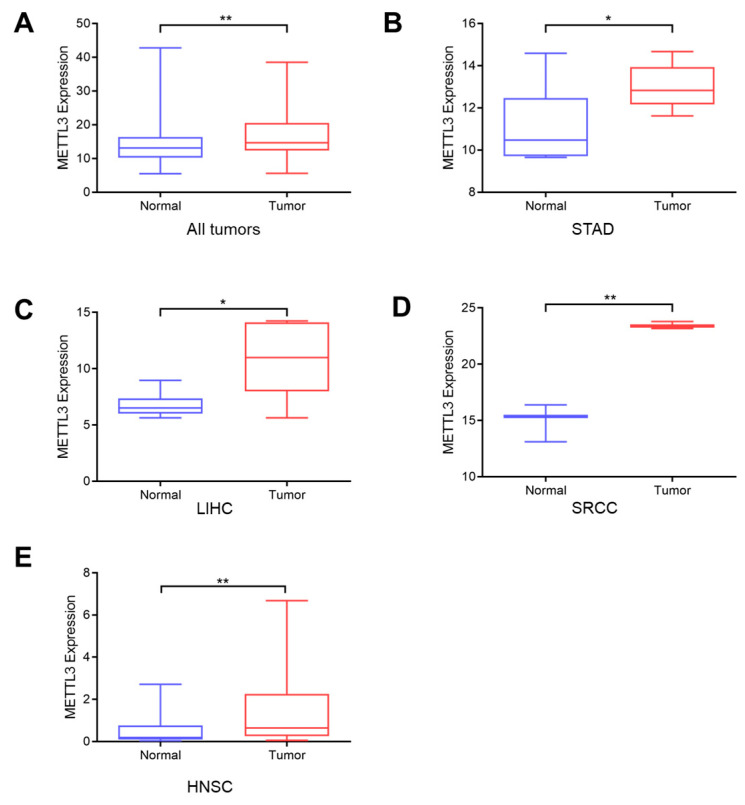
(**A**) Elevated expression levels of METTL3 in pan-cancer tumor tissues in GSE87410 (** *p* < 0.01). (**B**–**D**) Elevated expression levels of METTL3 in STAD, LIHC and SRCC tissues included in the GSE87410 dataset (* *p* < 0.05, ** *p* < 0.01). (**E**) Elevated mRNA levels of METTL3 in HNSC tissues in our collected tissues (** *p* < 0.01).

As shown in Figure 4A, the expression level of METTL3 was significantly higher in tumor tissues compared to that in adjacent normal tissues according to the analysis of all patients in the GEO87410 dataset. This result is consistent with the results of TCGA analyses, which also suggested that METTL3 was upregulated in pan-cancer tissues compared to that in matched normal tissues. We next investigated the expression of METTL3 in each type of cancer. The results suggested that METTL3 expression was significantly higher in tumor tissues than in normal tissues in STAD, LIHC, and SRCC (Figure 4B–D). These results are also consistent with those of TCGA analyses of STAD and LIHC.

**Figure 5:**
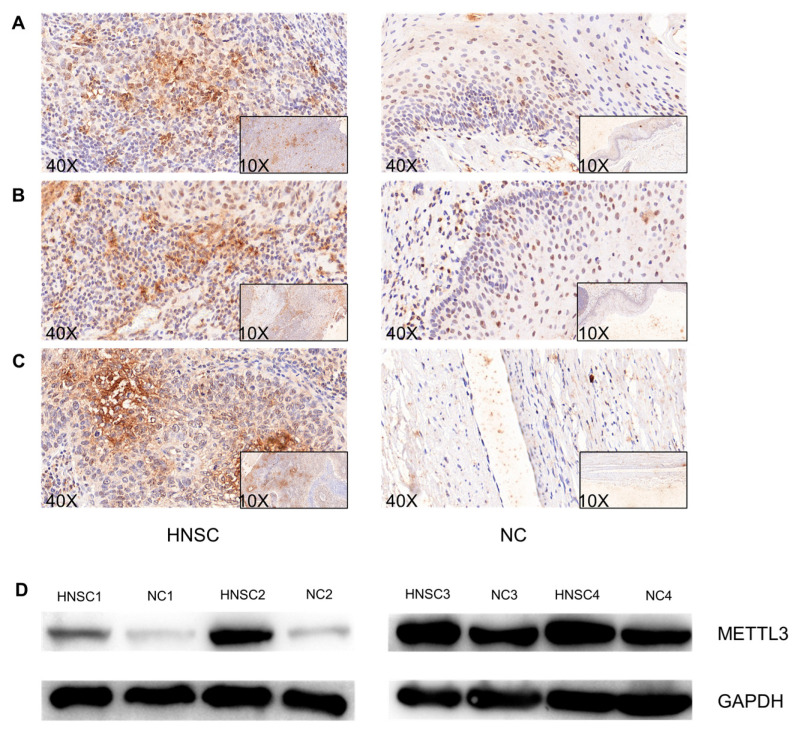
(**A**–**C**) Representative immunohistochemical images of elevated levels of METTL3 protein in HNSC tissues in our collected tissues. (**D**) Representative Western blot images of elevated levels of METTL3 protein in HNSC tissues in our collected tissues.

Furthermore, the quantitative reverse transcription-polymerase chain reaction indicated that METTL3 expression levels were markedly upregulated in HNSC tissues compared to those in adjacent normal tissues in our cohort (Figure 4E). Immunohistochemistry and Western blot analysis showed that METTL3 protein expression was significantly upregulated in HNSC tissues compared to that in normal tissues (Figure 5A–D). 

## 4. Discussion 

METTL3 is highly conserved across various species, from *Drosophila* to zebrafish and mice [25,26]. METTL3 regulates cerebellar development [27], spermatogonial differentiation [28], and various biological processes in different cancers [29]. For example, METTL3 plays essential roles in preserving glioma stem-like cells and radio-resistance by stabilizing the mRNA of m^6^A-modified SOX2 via HuR [30]. In colorectal cancer, METTL3 promotes tumorigenesis by activating the m^6^A–GLUT1–mTORC1 axis [31].

The expression profiles of METTL3 in pan-cancer indicate that METTL3 is upregulated in various cancers. Higher METTL3 expression levels are significantly associated with poor overall survival in ACC, KICH, and LIHC and poorer DFS in ACC, CESC, HNSC, KICH, and LIHC, supporting the potential of METTL3 as a prognostic biomarker in cancers, particularly in ACC, KICH, and LIHC. 

Multiple types of immune cells have been suggested to be associated with METTL3 expression in various cancers, especially T cells. In addition to tumor-infiltrating lymphocytes [32,33], TMB and MSI are regarded as potential predictors of immunotherapy effects [34,35,36,37]. Recognition of mutation-derived neoantigens by the immune system may generate an antitumor response and a neoantigen load with potent biomarkers for cancer immunotherapy [38,39]. Additionally, studies have indicated an association between clinical immune therapy effects and prognoses and the expression of immune checkpoint genes, including CTLA-4 and TIGIT, in various cancers [40,41]. Our results indicate that METTL3 expression is correlated with MMR gene expression in all types of cancers and correlated with the TMB, MSI, neoantigen loads, and immune checkpoint gene expression in certain cancer types. Thus, METTL3 may participate in regulation of the tumor immune microenvironment and may serve as a potential prognostic biomarker of the effects of immune therapies.

As for the PPI analysis of METTL3, METTL14 dramatically connected with METTL3 via physical interaction and predicted functional relationships. In the absence of catalytic activity, METTL14 can interact with METTL3 to recognize substrate RNAs and transfer methyl groups by forming a stable complex [16]. As the central component of the methyltransferase complex, dysregulated METTL14 is involved in the initiation and progression of various tumors [42]. 

WTAP is indispensable for the stabilization of METTL3 and METTL14 and plays essential roles in their localization into nuclear speckles [43]. WTAP was found to be highly expressed in LIHC, and WTAP expression may serve as an independent predictor of LIHC survival. WTAP can regulate m^6^A modification to suppress ETS1 post-transcriptionally, which was shown to modulate the cell cycle of LIHC cells via p21/p27-dependent patterns mediated by ETS1 [44]. 

METTL3 was enriched in pathways related to RNA modification and metabolism. This result is consistent with the fact that METTL3 regulates the m^6^A levels on RNA to modulate RNA metabolism [45]. By modulating the functions of oncogenes and tumor-suppressing genes via m^6^A modification, METTL3 plays essential roles in the development and progression of diverse cancers [46,47]. Furthermore, METTL3 can participate in the immune regulation of various cancers by affecting the m^6^A modification of different immune-related genes. However, METTL3 may play different roles in various cancers according to the different pathogenic and multistage carcinogenesis mechanisms of different cancer types. 

In pan-cancer analysis, EMT was significantly associated with METTL3 aberrant expression. This result is consistent with the results showing that the expression levels of METTL3 in patients with metastatic SKCM differed from that in patients with SKCM without metastasis. METTL3 may participate in EMT regulation by modulating m^6^A modifications [48]. During EMT, the levels of m^6^A modification of mRNA are increased, with METTL3 acting as the critical regulator [49]. Thus, METTL3 may be a therapeutic target in cancer treatment for inhibiting EMT and the metastasis of cancers.

Although the clinical prognostic values and regulatory roles of METTL3 were comprehensively analyzed, there were several limitations to our study. First, our results are based on bioinformatic analyses, and experimental studies are needed to validate our results. Second, the mRNA levels of METTL3 were used to explore its role in pan-cancer. However, the actions of functional proteins can be modulated by post-translational modifications [50]. Third, the specific mechanisms by which METTL3 influences the tumor immune microenvironment should be further evaluated.

## 5. Conclusions

The clinical prognostic value of METTL3 in different types of cancers was validated. METTL3 may play essential roles in modulating tumor immune responses and EMT pathways. METTL3 may serve as a potential prognostic biomarker and immune therapy target in clinical settings.

## Data Availability

The data presented in this study are available on request from the corresponding author.

## Supplementary Materials

The following supporting information can be downloaded at: https://www.mdpi.com/article/10.3390/jcm12010155/s1, Table S1. The abbreviations of various cancer types. Figure S1. Associations between METTL3 expression levels and tumor-infiltrating cells in LIHC, LUAD, PAAD, PRAD, SKCM and THCA. Figure S2. Correlation analysis between expression levels of METTL3 and the numbers of tumor neoantigens in pan-cancer. Figure S3. (A) Radar map of correlation analysis between METTL3 expression and TMB in pan-cancer. (B) Radar map of correlation analysis between METTL3 expression and MSI in pan-cancer. Figure S4. PPI network of METTL3 constructed with GeneMANIA. Figure S5. GO and KEGG pathway enrichment analysis of METTL3 and the genes involved in its PPI network. The size of the nodes indicated enriched the number of genes enriched in the terms; the color indicated the P values, the redder the color, the more significant it is. Figure S6. GSEA plots of different expression levels of METTL3. (A) The enriched KEGG terms with the high METTL3 expression samples. (B) The enriched KEGG terms with the low METTL3 expression samples. (C) The enriched HALLMARK terms with the high METTL3 expression samples. (D) The enriched HALLMARK terms with the low METTL3 expression samples. Figure S7. The expression levels of METTL3 in CESC, ESCA, LUAD, LUSC, THCA and SCLC tissues included in GSE87410.
