# Supplementary material for: Prognostic Values of METTL3 and Its Roles in Tumor Immune Microenvironment in Pan-Cancer
Source: J Clin Med. 2022 Dec 25;12(1):155. doi: 10.3390/jcm12010155 (PMC9821157; doi:10.3390/jcm12010155)
Supplement: Supplementary file 1 [file jcm-12-00155-s001.zip › jcm-2050015-supplementary.pdf]

Supplementary Material:

Supplementary Tables

Supplementary Table S1. The abbreviations of various cancer types.

Abbreviations	Cancers
**ACC**	Adrenocortical carcinoma
**BLCA**	Bladder Urothelial Carcinoma
**BRCA**	Breast invasive carcinoma
**CESC**	Cervical squamous cell carcinoma and endocervical adenocarcinoma
**CHOL**	Cholangio carcinoma
**COAD**	Colon adenocarcinoma
**DLBC**	Lymphoid Neoplasm Diffuse Large B-cell Lymphoma
**ESCA**	Esophageal carcinoma
**GBM**	Glioblastoma multiforme
**HNSC**	Head and Neck squamous cell carcinoma
**KICH**	Kidney Chromophobe
**KIRC**	Kidney renal clear cell carcinoma
**KIRP**	Kidney renal papillary cell carcinoma
**LAML**	Acute Myeloid Leukemia
**LGG**	Brain Lower Grade Glioma
**LIHC**	Liver hepatocellular carcinoma
**LUAD**	Lung adenocarcinoma
**LUSC**	Lung squamous cell carcinoma
**MESO**	Mesothelioma
**OV**	Ovarian serous cystadenocarcinoma
**PAAD**	Pancreatic adenocarcinoma
**PCPG**	Pheochromocytoma and Paraganglioma
**PRAD**	Prostate adenocarcinoma
**READ**	Rectum adenocarcinoma
**SARC**	Sarcoma
**SKCM**	Skin Cutaneous Melanoma
**STAD**	Stomach adenocarcinoma
**TGCT**	Testicular Germ Cell Tumors
**THCA**	Thyroid carcinoma
**THYM**	Thymoma
**UCEC**	Uterine Corpus Endometrial Carcinoma
**UCS**	Uterine Carcinosarcoma
**UVM**	Uveal Melanoma

Supplementary Figures

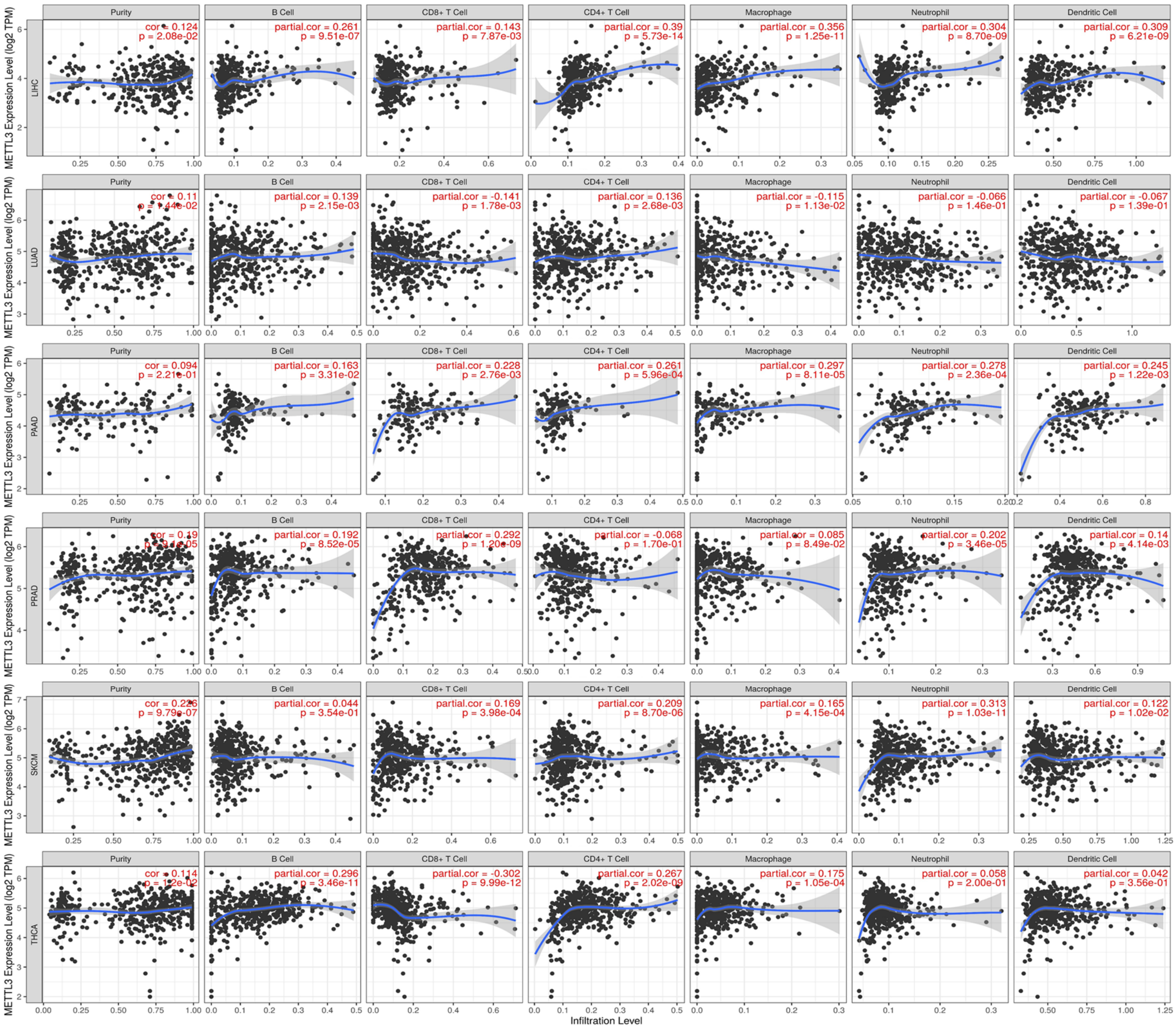

Supplementary Material Figure S1. Associations between METTL3 expression levels and tumor-infiltrating cells in LIHC, LUAD, PAAD, PRAD, SKCM and THCA.

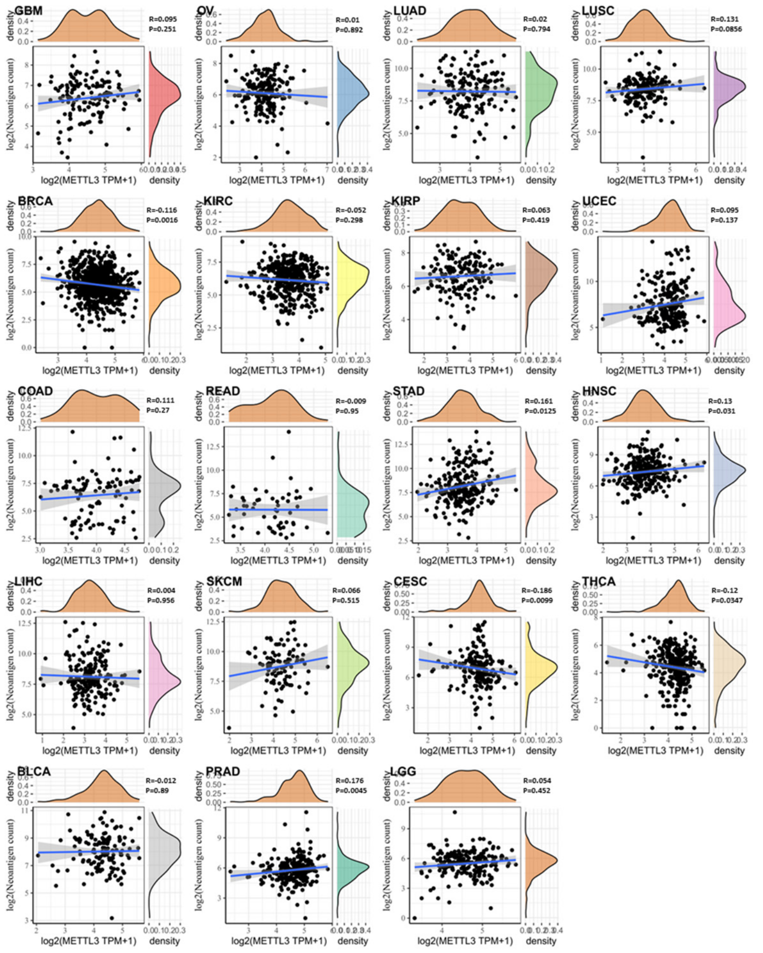

Supplementary Material Figure S2. Correlation analysis between expression levels of METTL3 and the numbers of tumor neoantigens in pan-cancer.

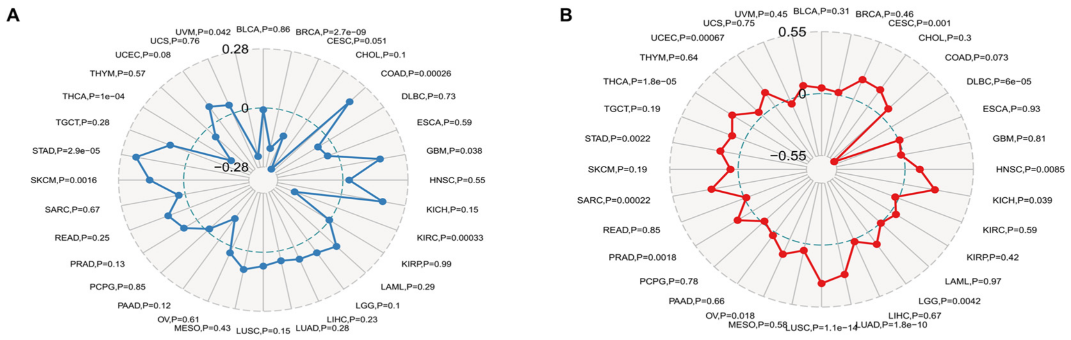

Supplementary Material Figure S3. (A) Radar map of correlation analysis between METTL3 expression and TMB in pan-cancer. (B) Radar map of correlation analysis between METTL3 expression and MSI in pan-cancer.

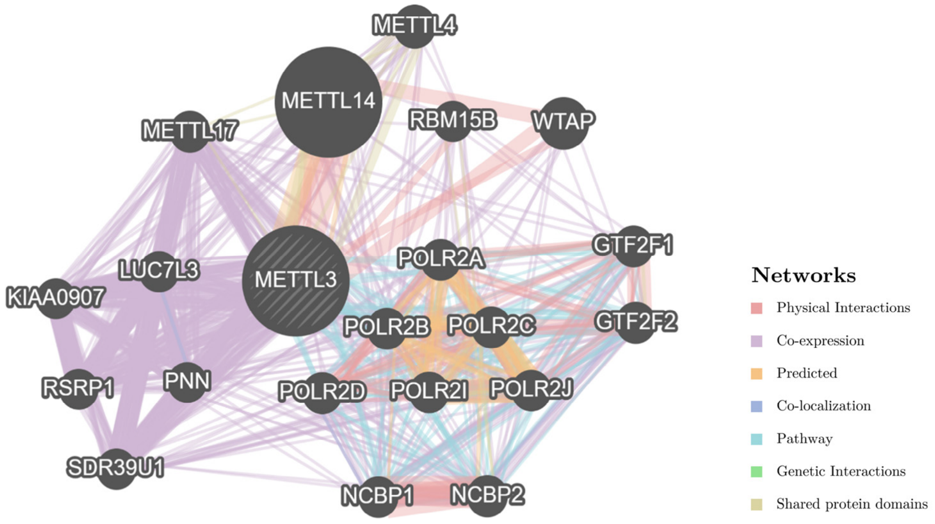

Supplementary Material Figure S4. PPI network of METTL3 constructed with GeneMANIA.

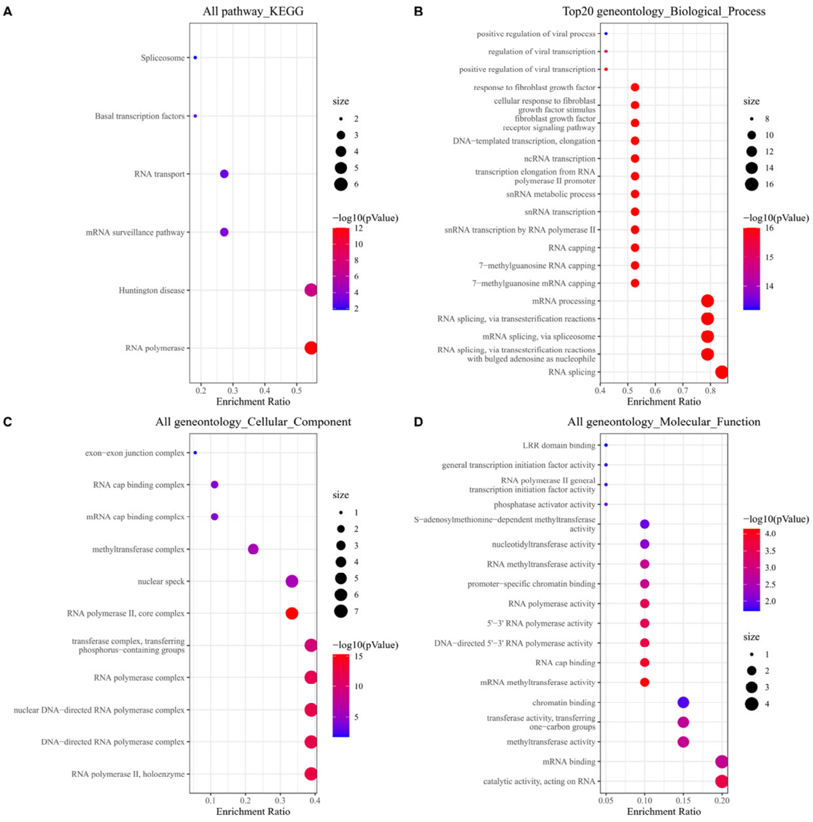

Supplementary Material Figure S5. GO and KEGG pathway enrichment analysis of METTL3 and the genes involved in its PPI network. The size of the nodes indicated enriched the number of genes enriched in the terms; the color indicated the P values, the redder the color, the more significant it is.

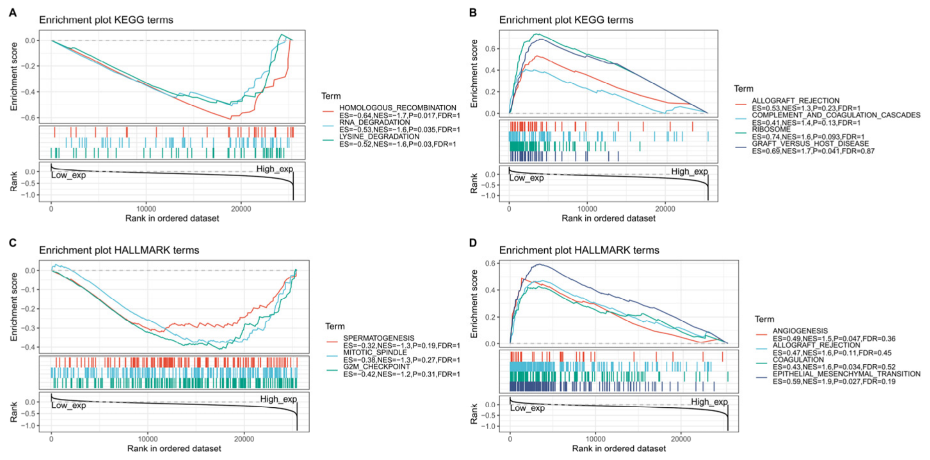

Supplementary Material Figure S6. GSEA plots of different expression levels of METTL3. (A) The enriched KEGG terms with the high METTL3 expression samples. (B) The enriched KEGG terms with the low METTL3 expression samples. (C) The enriched HALLMARK terms with the high METTL3 expression samples. (D) The enriched HALLMARK terms with the low METTL3 expression samples.

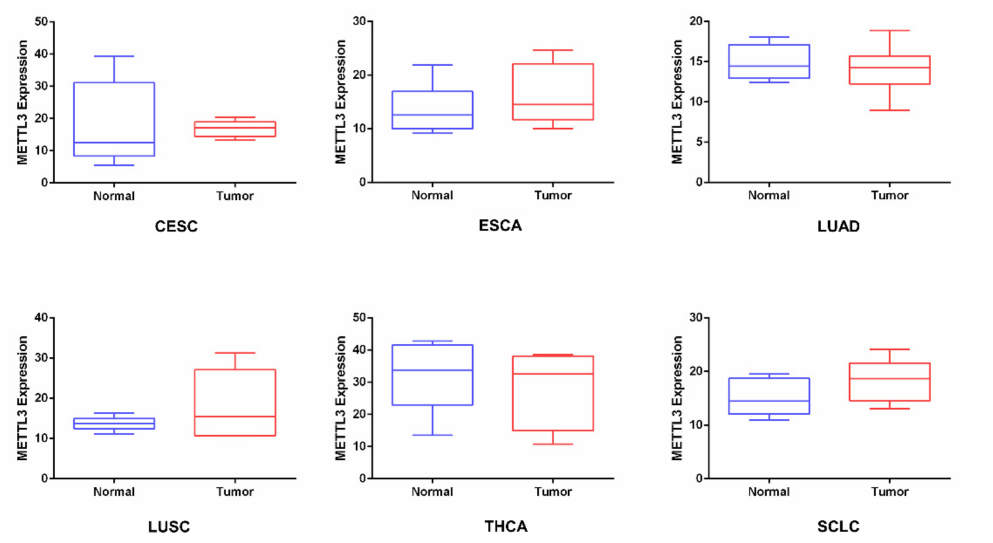

Supplementary Material Figure S7. The expression levels of METTL3 in CESC, ESCA, LUAD, LUSC, THCA and SCLC tissues included in GSE87410.
